# Authorship practices must evolve to support collaboration and open science

**DOI:** 10.1371/journal.pbio.3002364

**Published:** 2023-10-13

**Authors:** Veronique Kiermer

**Affiliations:** Public Library of Science, San Francisco, California, United States of America

## Abstract

Journal authorship practices have not sufficiently evolved to reflect how research is done. In this Perspective, Veronique Kiermer argues for urgent improvements to support teams, collaboration, and open science.

## Teaser

Journal authorship practices have not sufficiently evolved to reflect the way research is now done. Improvements to support teams, collaboration, and open science are urgently needed.

This article is part of the *PLOS Biology* 20th Anniversary Collection.

An author is understood in popular language to be the writer of a piece of work, its originator, or creator. In biology, being a research article author has acquired a specific meaning as the currency of credit for research work: It is the certification that one has contributed to the research and what ultimately counts when researchers are assessed. Tackling meaningful problems in biology increasingly requires multidisciplinary teams, in which individuals bring various skills, expertise, and resources. As the number of authors per article has increased, individual contributions—the focus of researcher assessment exercises—are obscured in long author lists. Authorship standards and digital infrastructure, still rooted in the original definitions, have not evolved to reflect the way research is done.

At a time when policy makers increasingly call for open science, many contributions that enable open science practice can be excluded altogether if they do not fit current authorship criteria. The consequences for establishing an open research culture and for encouraging important collaborations are real. For example, researchers in the Global South who have access to endemic pathogen isolates may understandably hesitate to share their sequences as too often they are not credited as authors when analyses—enabled by their data and conducted with the computational power and skills available in the Global North—are published. Preprints offer an opportunity to get early credit for such foundational data, but it is incumbent on those using the data to recognize these early contributors, as some have done in exemplary manner [1].

Even when all contributors are included, first and last authors are assigned particular value in research assessment contexts, but to understand the significance of other authors’ roles one needs to consult Author Contribution Statements. While these statements are now demanded by more journals, they are not always easily accessible. Assessors still rely on metrics, despite many calls for caution (e.g., [2]), and bibliometricians have developed mathematical tricks to weigh contributions as a function of position in the authorship list [3]. This sort of accounting creates a climate that is antithetical to team science, as it incentivizes collaborators to compete for prime positions.

Researcher assessment reform is urgently needed to align incentives and create a culture in which all meaningful contributions to research are recognized and rewarded. Of course, such reform will require long-term systemic changes for researchers, funders, and institutions, as well as for journals. For systemic changes to take hold, each agent in the system has a responsibility to start making small adjustments where they can be made. Considering the centrality of article authorship in the current research assessment frameworks, I would argue that journals and their systems need to improve to address fundamental problems and support an authorship culture that recognizes all meaningful contributions (Box 1).

**Box 1. Suggested improvements for journals to promote authorship practices aligned with open science and collaboration**

**Problem 1**

Authorship standards are rigid and not necessarily aligned with evolving best practice in the field. To address this, we need to:

- Reinforce authorship norms of credit for all meaningful contributions and accountability post-publication.
- Provide a framework to guide discussion and alignment between coauthors about credit and accountability.
- Require transparency of all authors’ contributions.

**Problem 2**

Author contributions are unclear and not easily discoverable. To address this, we need to:

- Capture each author’s contributions in standardized machine-readable ways (e.g., metadata implementation of the CRediT taxonomy).
- Develop infrastructure to enable the flow of authorship information to indexers.
- Facilitate the integration of author contributions information in research assessment systems.

**Problem 3**

Article publications are perceived to be the only valued output of research. To address this, we need to:

- Implement the use of persistent identifiers (PIDs) and contributor credit for non-article research outputs such as data, code, and methods by depositing them in specialized repositories or preprint servers.
- Promote citations of these outputs as bona fide academic outputs.
- Track their evolution post-publication and measure the value they add to the research community.

Journals authorship policies tend to dictate strict standards about the type and level of contributions that qualifies for authorship; however, this approach is challenging and, ultimately, can be counterproductive. There are inevitably field-specific variations in these determinations [4], and best practices are evolving. For example, contributions that are critical to making research data accessible and reusable by other researchers (a best practice increasingly required by funders) are not, in and of themselves, recognized as worthy of authorship by one commonly adopted authorship standard—the International Committee of Medical Journal Editors (ICMJE) Authorship Requirements)—but they are recognized by a more recent guideline that expands the ICMJE Requirements to align them with multiple disciplines and with team science [5]. Most of the PLOS portfolio, including *PLOS Biology*, has adopted this more inclusive guideline, which allows the inclusion of authors who do not report drafting or editing the article but instead make important contributions like data analysis, data curation, methodology, or software development. A rigid standard that requires drafting or editing the article as a sine qua non criteria, like the ICMJE Requirements, would exclude these important contributors from the authors list.

Instead of trying to enforce rigid standards, journals can help reinforce norms of authorship and encourage discussion and alignment between researchers. Importantly, being an author is not only about credit but also about accountability. Typically, an author will be accountable for the quality and integrity of their own contribution, but also for the work as a whole by ensuring that questions arising post-publication are investigated thoroughly and that materials and data remain available. Journals can reinforce these norms by clearly communicating expectations of accountability and by providing guidelines as a framework to guide discussion between coauthors. Initiating discussions about authorship early in the project, and revisiting them to account for changing circumstances, is one of the strategies that can support fair authorship practices [6].

To avoid inappropriate practices, such as guest and honorary authorship, relaxing authorship requirements must go hand in hand with increasing transparency. Public, transparent, and easily processed disclosures of authors’ contributions have a normative role. They showcase credit for relevant types of contributions and support accountability after publication. In a best case scenario, when an element of research work is particularly useful for the research community, prospective users and collaborators know who to contact and who to credit. In a worst case scenario, when allegations of irregularities are made, research integrity officers can identify individual contributions to help focus investigations and limit indiscriminate repercussions.

But even if journals ensure that all meaningful contributions are included and described transparently, it does not automatically follow that research assessors, for example, in tenure and promotion committees, will pay attention. This information must be surfaced through the digital infrastructure of scholarly communication to be discoverable, readily accessible, and integrated in research assessment frameworks. The CRediT taxonomy was developed as a human- and machine-readable taxonomy of researchers’ contributions to facilitate such discoverability and has been adopted by thousands of journals [7]. The US National Information Standards Organization (NISO) has now made it a standard and is acting as a steward to ensure the taxonomy evolves to meet researchers’ needs and is understood consistently [8]. More journals should adopt CRediT and the digital infrastructure should support the flow of CRediT metadata to ORCID records and to indexers like PubMed and Google Scholar.

With these fundamental elements in place, there will be multiple ways for authorship to evolve. Some have argued for abandoning the concept of authorship in favor of a contributorship model, whereby all contributors are listed and their contributions identified via CRediT terms [9]. The idea has substantial value but faces social resistance, as the notion of authorship is so engrained. I have concerns that an incomplete shift, in which authors and contributors coexist in a two-tier system, would ultimately formalize inequities. Recently, others have proposed micro-citations that would allow every claim in a research article to be traced to an author, either through an adaptation of the CRediT taxonomy called MeRIT [10], or through textual conventions [11]. Further experimentation is needed to test the effectiveness of micro-citations, but without better metadata flow, they may simply remain invisible.

The centrality of article authorship in recognizing research contributions is such that specialized article types have been developed to describe datasets, software, or protocols. However, these are not always the most effective format for research outputs that, by definition, need to be updated with practice. Ultimately, we need research assessment reform to move beyond recognizing only articles and to value contributions such as data, software, and methodologies as bona fide academic outputs. Journals can support that transition by promoting the consistent use of persistent identifiers and citations for these other research outputs [12–14], and by monitoring their usefulness for the research community. Importantly, some contributions that will enhance the usefulness of these resources, like improving datasets, code packages, and laboratory protocols, occur after publication. We need new ways of recording these activities to be able to value them.

It does not serve us well to reduce everything to articles as the only valuable output of research. But the current reality is that authorship of articles is central to research assessment, and while research assessment reform starts to take hold, journals can and should improve their authorship practices to ensure that all important contributions to how science is done today can be recognized and valued. This is essential for team science, open science, and equitable collaboration to flourish.
